# Fermented *Protaetia brevitarsis* Larvae Improves Neurotoxicity in Chronic Ethanol-Induced-Dementia Mice via Suppressing AKT and NF-κB Signaling Pathway

**DOI:** 10.3390/ijms25052629

**Published:** 2024-02-23

**Authors:** Hyo Lim Lee, Jong Min Kim, Min Ji Go, Han Su Lee, Ju Hui Kim, Ho Jin Heo

**Affiliations:** Division of Applied Life Science (BK21), Institute of Agriculture and Life Science, Gyeongsang National University, Jinju 52828, Republic of Korea; gyfla059@gnu.ac.kr (H.L.L.); myrock201@gnu.ac.kr (J.M.K.); alswl9245@gnu.ac.kr (M.J.G.); ns3005@naver.com (H.S.L.); zkfkapflove@nate.com (J.H.K.)

**Keywords:** cognitive function, alcoholic dementia, edible insects, neuroinflammation, synaptic plasticity

## Abstract

This study was investigated to examine the neuroprotective effect of fermented *Protaetia brevitarsis* larvae (FPB) in ethanol-induced-dementia mice. Consumption of FPB by mice resulted in improved memory dysfunction in the Y-maze, passive avoidance, and Morris water maze tests. FPB significantly decreased oxidative stress by regulating levels of malondialdehyde (MDA), superoxide dismutase (SOD), and reduced glutathione (GSH) in brain tissues. In addition, FPB restored cerebral mitochondrial dysfunction by modulating levels of reactive oxygen species (ROS), mitochondrial membrane potential (MMP), and ATP. In addition, FPB enhanced the cholinergic system via the regulation of acetylcholine (ACh) content, acetylcholinesterase (AChE) activity, and expressions of AChE and choline acetyltransferase (ChAT) in brain tissues. FPB ameliorated neuronal apoptosis through modulation of the protein kinase B (AKT)/B-cell lymphoma (BCL)-2 signaling pathway. Also, FPB improved inflammation response by down-regulating the toll-like receptor (TLR)-4/nuclear factor (NF)-κB pathway. Additionally, FPB ameliorated synaptic plasticity via the increase of the expressions of synaptophysin (SYP), postsynaptic density protein (PSD)-95, and growth-associated protein (GAP)-43. Treatment with FPB also reinforced the blood–brain barrier by increasing tight junctions including zonula occludens (ZO)-1, occludin, and claudin-1. In conclusion, these results show that FPB can improve cognitive impairment via AKT/NF-κB pathways in ethanol-induced-dementia mice.

## 1. Introduction

Alcohol is a toxic and psychoactive substance; dependence on alcohol can affect structures and processes in the central nervous system [1]. Excessive consumption of alcohol can induce structural and functional disorders of the central nervous system and increase the risk of alcohol-related dementia (ARD) [2]. Because the diagnostic criteria for ARD are vague, prevalence figures are difficult to establish. However, according to previous studies, the prevalence of ARD is estimated to account for 9–22% of all dementia subtypes [2]. More than 90% of alcohol in the body undergoes oxidation and metabolism in the liver and produces acetaldehyde and excessive ROS, which are known to be toxic to the central nervous system [3]. As a result, metabolization of alcohol in the body can induce a chain reaction of reactive oxygen species (ROS) and lipid peroxidation, resulting in damage to the brain [3]. Excessive accumulation of ROS can also cause mitochondrial dysfunction by decreasing mitochondrial membrane potential (MMP) and reducing the production efficiency of ATP [4]. Consequently, accumulation of ROS within mitochondria can lead to brain neuronal cell death through activation of apoptosis signaling [5]. In addition, alcohol consumption can induce leakage of lipopolysaccharide (LPS) derived from Gram-negative bacteria, leading to increased intestinal permeability as well as systemic inflammation [1]. LPS transport mainly occurs through receptors such as the toll-like receptor (TLR)-4, and TLR signaling is known to induce an inflammatory response through the activation of nuclear factor (NF)-κB [6]. This is followed by the generation of inflammatory cytokines such as tumor necrosis factor (TNF)-α and interleukin (IL)-1β, which can cross the blood–brain barrier (BBB), contributing to alcoholic neuroinflammation [7]. Increased synergy due to the accumulation of ROS and inflammatory responses in the brain can eventually lead to the development of neurotoxicity. In addition, ethanol-based neural toxicity and the resulting apoptosis are likely involved in cholinergic neurotransmission [8]. Cholinergic dysfunction caused by a decrease in the levels of acetylcholine (ACh) and an increase in the levels of acetylcholine esterase (AChE) can cause neuronal signaling disorders and contribute to memory decline [9]. Thus, stimulation of neuroinflammatory processes by alcohol toxicity and oxidative stress can induce neuronal apoptosis and changes in neuroplasticity, ultimately affecting learning and memory abilities.

Insects are a natural resource that have been utilized for various purposes as ingredients in food and medicine since ancient times [10]. Due to explosive population growth worldwide, edible insects, which are considered nutritionally excellent food materials, have been regarded as a next-generation alternative food source [10,11]. However, despite the conducting of nutritional and bio-functional research using various edible insects, industrialization has not been attempted due to consumer aversion [6,10]. Therefore, conducting of research to enhance the bioactivity of edible insects will be required in the effort to enhance their value as a functional food material.

As a representative species of edible insects, *Protaetia brevitarsis* (*P. brevitarsis*) larvae, a member of the white-spotted beetle family, have been reported to exert effects of neuroprotection, antioxidation, and anti-inflammation [12,13,14]. In addition, *P. brevitarsis* larvae contain an abundance of proteins and fats, particularly a variety of essential amino acids and unsaturated fatty acids [15]. Various studies analyzing the nutritional components and activity of *P. brevitarsis* larvae using different pretreatments including solvent extraction, protein hydrolysis, and beneficial bacteria fermentation have been reported [16]. Microorganisms produce beneficial nutrients including minerals, vitamins, and organic acid, and therefore can also be used for enhancement of functional substances [17] In addition, fermentation of *Locusta migratoria* and *Galleria mellonella* with beneficial bacteria has been reported to result in increased levels of lactic acid, free fatty acids, and free amino acids [15]. However, few studies using *P. brevitarsis* larvae fermented with *Bacillus subtilis* (*B. subtilis*) have been reported. In our previous study, *P. brevitarsis* larvae were fermented with the beneficial microorganism *B. subtilis* to increase their utility as food and to increase the amino acid content [18]. The results confirmed that the content of free amino acids, including essential amino acids, increased compared to another previously reported study [19]. However, research on the mechanism of the protective effect against ethanol-induced dementia using edible insects fermented with useful microorganisms is still insufficient. Therefore, the objective of this study was to confirm the neuroprotective effect of fermented *P. brevitarsis* larvae (FPB) on ethanol-induced dementia in mice.

## 2. Results

### 2.1. Cytoprotective Effect of FPB on Ethanol and H_2_O_2_-Induced HT22 Cells

#### 2.1.1. Cell Viability

**Figure 1:**
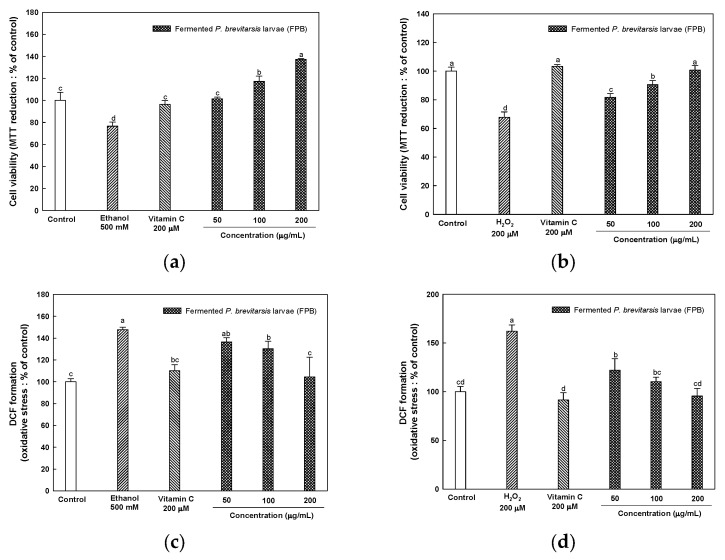
Cytoprotective effect of fermented *Protaetia brevitarsis* larvae (FPB) in ethanol and H_2_O_2_-induced cell viability (**a**,**b**) and oxidative stress level (**c**,**d**) in HT22 cells. Data were represented as mean ± SD (*n* = 5). Values with different superscripts on the bar graph indicate statistical differences (*p* < 0.05).

To evaluate the protective effect of FPB against ethanol and H_2_O_2_-induced HT22 cells, cell viability was detected by a 3-(4,5-Dimethylthiazol-2-yl)-2,5-diphenyltetrazolium bromide (MTT) assay. As shown in Figure 1a,b, the cell viability of ethanol (76%) and H_2_O_2_ (67%) treated groups as the negative group was decreased compared to the control group (100%). However, the FPB treated group (137% and 100%, respectively) increased at 200 μg/mL compared to the negative groups. The cell viability of vitamin C as the positive control group was 96% and 103%, respectively.

#### 2.1.2. Intracellular Oxidative Stress

To evaluate the protective effect of FPB against ethanol and H_2_O_2_-induced HT22 cells, the intracellular oxidative stress was determined by a dichloro-dihydro-fluorescein diacetate (DCF-DA) assay. As shown in Figure 1c,d, the intracellular ROS level of ethanol- (147%) and H_2_O_2_- (162%) treated groups as the negative group was increased compared to the control group (100%). However, the FPB treated group (104% and 95%, respectively) increased at 200 μg/mL compared to the ethanol group as the negative groups. The intracellular oxidative stress level of vitamin C as the positive control group was 110% and 91%, respectively.

### 2.2. Effect of FPB on Behavioral Disorder in Ethanol-Administered Mice

#### 2.2.1. Y-Maze Test

**Figure 2:**
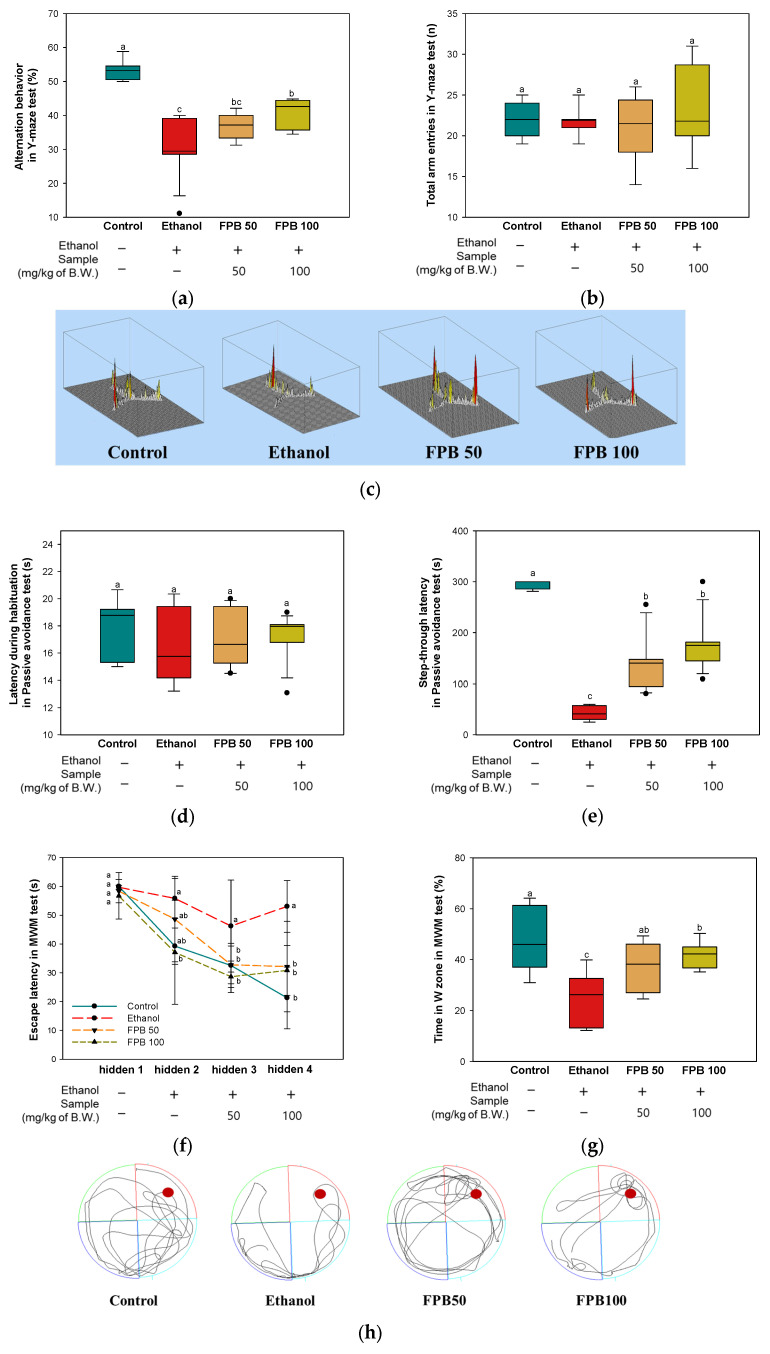
Effect of fermented *Protaetia brevitarsis* larvae (FPB) on behavioral disorder in ethanol-induced-dementia mice. Number of arm entries (**a**), alternation behavior (**b**), and 3D image of path tracking (**c**) in Y-maze test. Latency during habituation (**d**) and step-through latency (**e**) in passive avoidance test. Hidden platform test (**f**), probe test (**g**), and swimming pattern visualization image on probe test (**h**) in Morris water maze test. Data were represented as mean ± SD (*n* = 7). Values with different superscripts on the bar graph indicate statistical differences (*p* < 0.05).

To assess the effect of FPB on short-term spatial recognition memory ability, a Y-maze test was performed. As shown in Figure 2a, the number of arm entries showed no significant difference between all groups. The alternation behavior of the ethanol group (29.5%) decreased compared to the control group (53%). However, the FPB groups (FPB 50, 36%; FPB 100, 40%) effectively improved alternation behavior compared to the ethanol group (Figure 2b,c).

#### 2.2.2. Passive Avoidance Test

To confirm the effect of FPB on fear-motivated short-term learning and memory abilities, a passive avoidance test was conducted. As shown in Figure 2d, no significant difference in the time to enter the dark chamber from the light chamber was observed in any of the groups on the first day. On the second day, decreased step-through latency was observed in the ethanol group (39 s) compared with the control group (289 s). In contrast, increased step-through latency was observed in the FPB groups (FPB 50, 139 s; FPB 100, 176 s) compared with the ethanol group (Figure 2e).

#### 2.2.3. Morris Water Maze (MWM) Test

To evaluate the effect of FPB on long-term spatial recognition memory ability, MWM test was performed. As shown in Figure 2f, there was no significant difference in escape latency in any of the groups on the first day. The latency in finding the waterlogged platform of all groups decreased day by day, but the ethanol group was significantly longer than the other groups. In the hidden fourth days, the ethanol group (53 s) showed increased escape latency compared to the control group (21 s). However, the escape latency was ameliorated in the FPB groups (FPB50, 32 s; FPB 100, 30 s) compared to the ethanol group. In the probe test, the ethanol group (25%) spent less time compared to the control group (47%) in the W zone where the platform was located (Figure 2g,h). In contrast, the FPB groups (FPB 50, 37%; FPB 100, 41%) showed increased time spent in the W zone compared to the ethanol group.

### 2.3. Effect of FPB on Antioxidant Parameters in Ethanol-Administered Mice

#### 2.3.1. Malondialdehyde (MDA) Content

**Figure 3:**
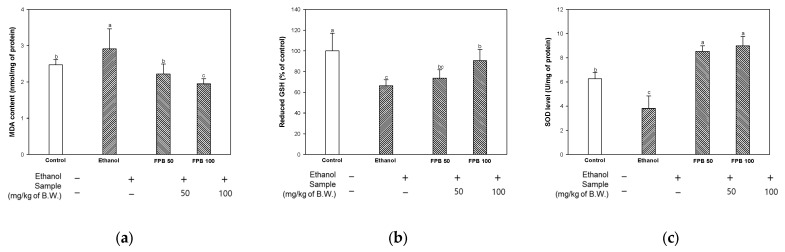
Effect of fermented *Protaetia brevitarsis* larvae (FPB) on antioxidant parameters in ethanol-induced-dementia mice. Malondialdehyde (MDA) production (**a**), reduced glutathione (GSH) (**b**), and superoxide dismutase (SOD) level (**c**) in brain tissues. Data were represented as mean ± SD (*n* = 5). Values with different superscripts on the bar graph indicate statistical differences (*p* < 0.05).

To estimate the effect of FPB on the antioxidant system, MDA content was detected. As shown in Figure 3a, the MDA content was increased in the ethanol group (2.9 nmol/mg of protein) compared to the control group (2.4 nmol/mg of protein). However, the MDA content was decreased in the FPB groups (FPB 50, 2.2 nmol/mg of protein; FPB 100, 1.9 nmol/mg of protein) compared to the ethanol group.

#### 2.3.2. Reduced Glutathione (GSH) Level

To evaluate the effect of FPB on the antioxidant system, reduced GSH level was measured. As shown in Figure 3b, the reduced GSH level was decreased in the ethanol group (66%) compared to the control group (100%). However, the reduced GSH level was increased in the FPB groups (FPB 50, 73%; FPB 100, 90%) compared to the ethanol group.

#### 2.3.3. Superoxide Dismutase (SOD) Level

To confirm the effect of FPB on the antioxidant system, SOD level was determined. As shown in Figure 3c, the SOD level was decreased in the ethanol group (3.8 U/mg of protein) compared to the control group (6.2 U/mg of protein). However, the SOD level was increased in the FPB groups (FPB 50, 8.5 U/mg of protein; FPB 100, 8.9 U/mg of protein) compared to the ethanol group.

### 2.4. Effect of FPB on Cholinergic System in Ethanol- Mice

#### 2.4.1. ACh Content

**Figure 4:**
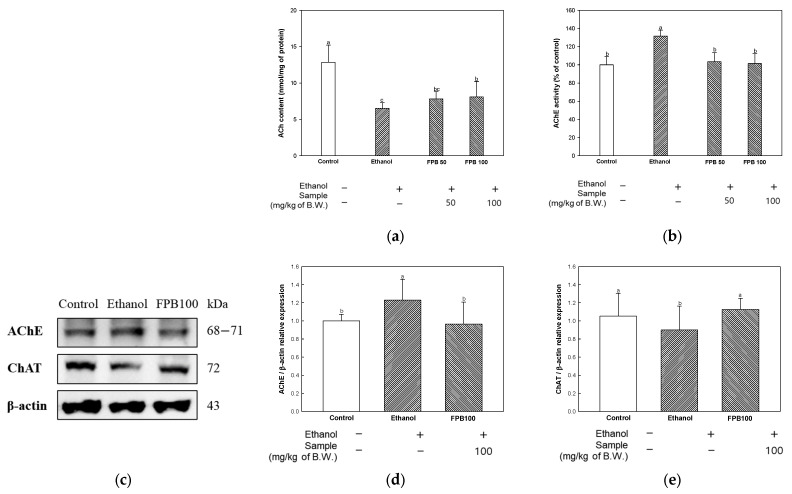
Effect of fermented *Protaetia brevitarsis* larvae (FPB) on cholinergic system in ethanol-induced-dementia mice. ACh content (**a**) and AChE activity (**b**) in brain tissues. Western blot band images (**c**) and the expression levels of AChE (**d**) and ChAT (**e**) in brain tissues. Data were represented as mean ± SD (ex-vivo, *n* = 5; Western blot, *n* = 3). Values with different superscripts on the bar graph indicate statistical differences (*p* < 0.05).

To assess the effect of FPB on the cholinergic system, ACh content was measured. As shown in Figure 4a, the ACh content was decreased in the ethanol group (6.5 nmol/mg of protein) compared to the control group (12.8 nmol/mg of protein). However, the ACh content was decreased in the FPB groups (FPB 50, 7.8 nmol/mg of protein; FPB 100, 8.1 nmol/mg of protein) compared to the ethanol group.

#### 2.4.2. AChE Activity

To estimate the effect of FPB on the cholinergic system, AChE activity was detected. As shown in Figure 4b, the AChE activity was increased in the ethanol group (131%) compared to the control group (100%). However, the AChE activity was decreased in the FPB groups (FPB 50, 103%; FPB 100, 101%) compared to the ethanol group.

#### 2.4.3. Protein Expression Levels of AChE and Choline Acetyltransferase (ChAT)

To confirm the effect of FPB on the cholinergic system, the expression levels of AChE and ChAT were determined using Western blot. As shown in Figure 4c,d, the relative expression level of AChE was increased in the ethanol group (1.22) compared to the control group (1.00). In contrast, the relative expression level of AChE was decreased in the FPB 100 group (0.96) compared to the ethanol group. The relative expression level of ChAT was decreased in the ethanol group (0.89) compared to the control group (1.00) (Figure 4c,e). However, the relative expression level of ChAT was increased in the FPB 100 group (1.12) compared to the ethanol group.

### 2.5. Effect of FPB on Mitochondrial Function in Ethanol-Administered Mice

#### 2.5.1. ROS Production

**Figure 5:**
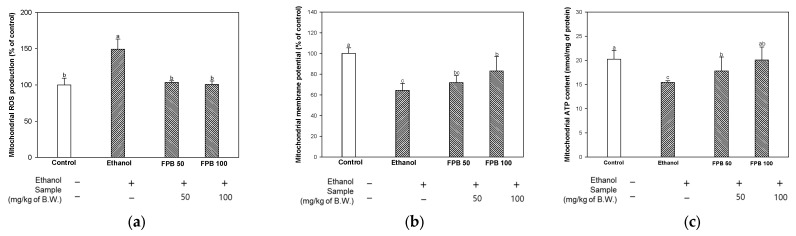
Effect of fermented *Protaetia brevitarsis* larvae (FPB) on mitochondrial function in ethanol-induced-dementia mice. Mitochondrial reactive oxygen species (ROS) production (**a**), mitochondrial membrane potential (MMP) (**b**), and mitochondrial ATP content (**c**) in brain tissues. Data were represented as mean ± SD (*n* = 5). Values with different superscripts on the bar graph indicate statistical differences (*p* < 0.05).

To assess the effect of FPB on mitochondrial function, the ROS level was measured. As shown in Figure 5a, mitochondrial ROS production was increased in the ethanol group (149%) compared to the control group (100%). However, ROS production was decreased in the FPB groups (FPB 50, 103%; FPB 100, 100%) compared to the ethanol group.

#### 2.5.2. MMP

To estimate the effect of FPB on mitochondrial function, MMP was detected. As shown in Figure 5b, the MMP was decreased in the ethanol group (64%) compared to the control group (100%). However, the MMP was increased in the FPB groups (FPB 50, 71%; FPB 100, 83%) compared to the ethanol group.

#### 2.5.3. ATP Content

To evaluate the effect of FPB on mitochondrial function, ATP content was determined. As shown in Figure 5c, mitochondrial ATP content was decreased in the ethanol group (15.4 nmol/mg of protein) compared to the control group (20.2 nmol/mg of protein). However, ATP content was increased in the FPB groups (FPB 50, 17.8 nmol/mg of protein; FPB 100, 20.1 nmol/mg of protein) compared to the ethanol group.

### 2.6. Effect of FPB on Neurotoxicity in Ethanol-Administered Mice

**Figure 6:**
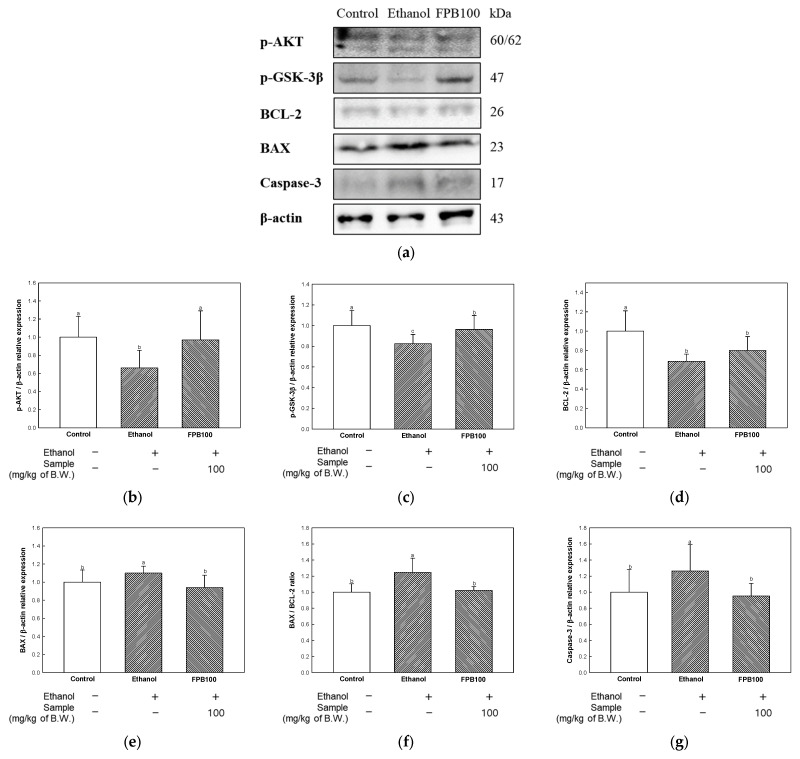
Effect of fermented *Protaetia brevitarsis* larvae (FPB) on neurocytotoxicity in ethanol-induced-dementia mice. Western blot band images (**a**), relative expression levels of p-AKT (**b**), p-GSK-3β (**c**), BCL-2 (**d**), BAX (**e**), BAX/BCL-2 ratio (**f**), and Caspase-3 (**g**) in brain tissues. Data were represented as mean ± SD (*n* = 3). Values with different superscripts on the bar graph indicate statistical differences (*p* < 0.05).

To confirm the effect of FPB on neurotoxicity, apoptosis-related markers were detected by Western blot. As shown in Figure 6a–d, the relative expression levels of phospho-protein kinase B (p-AKT), p-glycogen synthase kinase (GSK)-3β, and B-cell lymphoma (BCL)-2 were reduced in the ethanol group (0.65, 0.82, and 0.68, respectively) compared to the control group (1.00). However, the expression levels of p-AKT and p-GSK-3β were reduced in the FPB 100 group (0.96 and 0.96, respectively) compared to the ethanol group. The expression level of BCL-2 showed no statistically significant difference between the ethanol group and the FPB 100 group. The expression level of BCL associated X (BAX) and BAX/BCL-2 ratio were increased in the ethanol group (1.10 and 1.24, respectively) compared to the control group (Figure 6e,f). In contrast, the expression level of BAX and BAX/BCL-2 ratio were decreased in the FPB 100 group (0.94 and 1.02, respectively) compared to the ethanol group.

### 2.7. Effect of FPB on Neuroinflammation in Ethanol-Administered Mice

**Figure 7:**
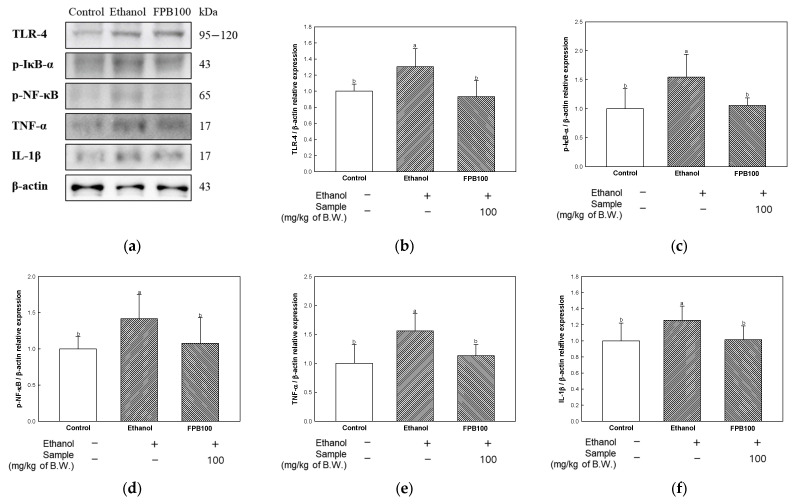
Effect of fermented *Protaetia brevitarsis* larvae (FPB) on neuroinflammation in ethanol-induced-dementia mice. Western blot band images (**a**), relative expression levels of TLR-4 (**b**), p-IκB-α (**c**), p-NF-κB (**d**), TNF-α (**e**), and IL-1β (**f**) in brain tissues. Data were represented as mean ± SD (*n* = 3). Values with different superscripts on the bar graph indicate statistical differences (*p* < 0.05).

To estimate the effect of FPB on neuroinflammation, TLR-4/NF-κB signaling pathway markers were detected by Western blot. As shown in Figure 7, the relative expression levels of TLR-4, p-NF-κB inhibitor (IκB)-α, p-NF-κB, TNF-α, and IL-1β were increased in the ethanol group (1.34, 1.54, 1.41, 1.56, and 1.25, respectively) compared to the control group (1.00). However, the expression levels of TLR-4, p-IκB-α, p-NF-κB, TNF-α, and IL-1β were reduced in the FPB 100 group (0.93, 1.05, 1.07, 1.13, and 1.01, respectively) compared to the ethanol group.

### 2.8. Effect of FPB on Neuroplasticity in Ethanol-Administered Mice

**Figure 8:**
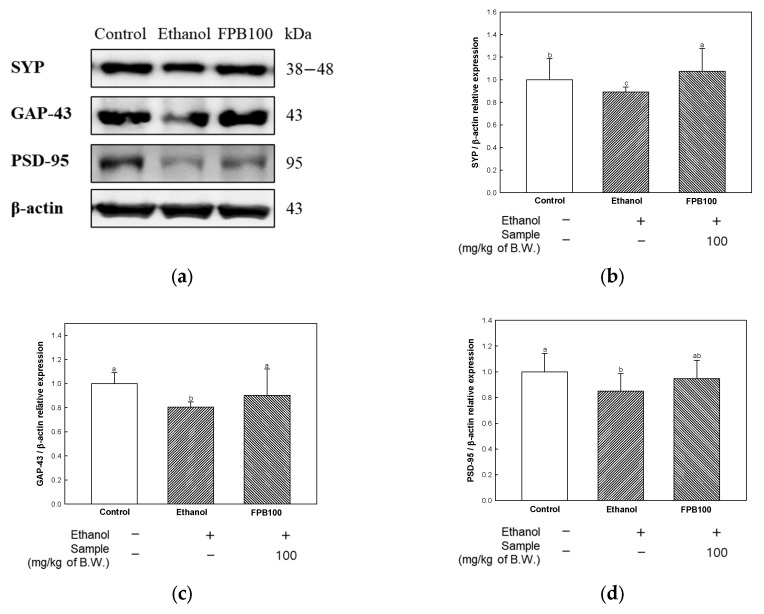
Effect of fermented *Protaetia brevitarsis* larvae (FPB) on synaptic protein markers in ethanol-induced-dementia mice. Western blot band images (**a**), relative expression levels of SYP (**b**), GAP-43 (**c**), and PSD-95 (**d**) in brain tissues. Data were represented as mean ± SD (*n* = 3). Values with different superscripts on the bar graph indicate statistical differences (*p* < 0.05).

To evaluate the effect of FPB on neuroplasticity, synaptic protein markers were detected by Western blot. As shown in Figure 8, the relative expression levels of synaptophysin (SYP), growth associated protein (GAP)-43, and postsynaptic density protein (PSD)-95 were decreased in the ethanol group (0.89, 0.80, and 0.84, respectively) compared to the control group (1.00). However, the expression levels of SYP, GAP-43, and PSD-95 were increased in the FPB 100 group (1.07, 0.90, and 0.94, respectively) compared to the ethanol group.

### 2.9. Effect of FPB on BBB Function in Ethanol-Administered Mice

**Figure 9:**
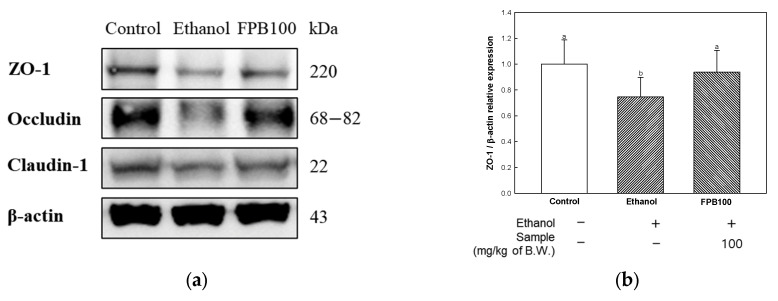

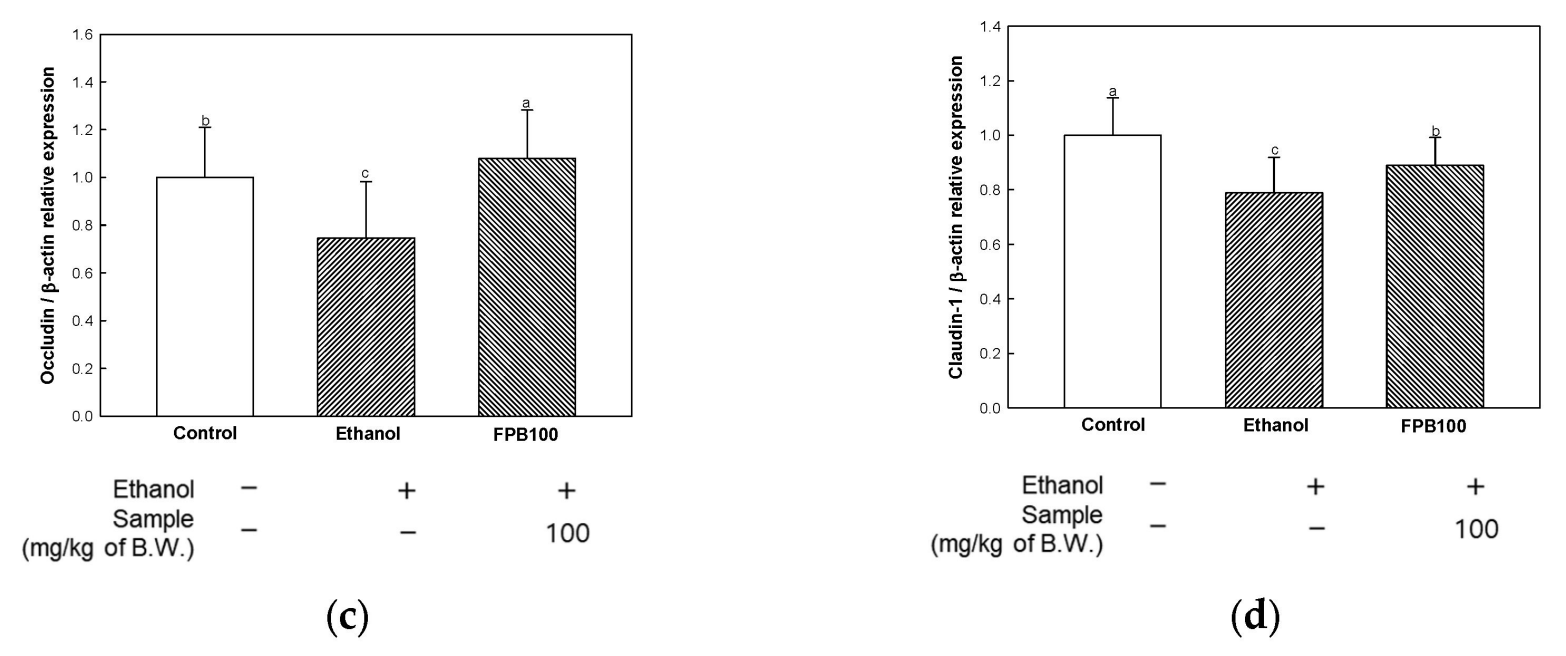
Effect of fermented *Protaetia brevitarsis* larvae (FPB) on blood–brain barrier (BBB) function in ethanol-induced-dementia mice. Western blot band images (**a**), relative expression levels of ZO-1 (**b**), occludin (**c**), and claudin-1 (**d**) in brain tissues. Data were represented as mean ± SD (*n* = 3). Values with different superscripts on the bar graph indicate statistical differences (*p* < 0.05).

To confirm the effect of FPB on BBB function, tight junction proteins were measured by Western blot. As shown in Figure 9, the relative expression levels of zonula occludens (ZO)-1, occludin, and claudin-1 were decreased in the ethanol group (0.74, 0.74, and 0.78, respectively) compared to the control group (1.00). However, the expression levels of ZO-1, occludin, and claudin-1 were increased in the FPB 100 group (0.93, 1.07, and 0.88, respectively) compared to the ethanol group.

### 2.10. Fatty Acid Composition of FPB

As shown in Table 1, eight types of fatty acids were detected in FPB. According to the results, the contents of saturated fatty acids, including myristic acid, palmitic acid, stearic acid, and arachidic acid, were 5.30, 143.80, 24.17, and 4.80 mg/100 g, respectively. The contents of monounsaturated fatty acids (MUFA) detected, including palmitoleic acid and oleic acid, were 48.67 and 357.07 mg/100 g. Polyunsaturated fatty acids (PUFA) detected in FPB included linoleic acid and linolenic acid at 6.00 and 4.80 mg/100 g.

## 3. Discussion

An association of chronic excessive alcohol consumption with multiple health problems including high blood pressure, diabetes, heart disease, and alcoholic liver disease has been reported [1]. One such complication, ARD, can be caused by both direct alcohol toxicity as well as indirect influences [2]. Unfortunately, few studies focusing on ARD have been reported, and only a few medications have been approved for treatment: disulfiram, acamprosate, and naltrexone [20]. However, these drug treatments have been associated with liver and kidney toxicity, making them unsuitable for long-term treatment. In addition, although mainly plant-derived ingredients have been discussed in the effort to demonstrate the effect on the improvement of ARD, research on edible insects has been limited. Thus, the current study examined the effect of FPB on the improvement of cognitive impairment in mice with chronic ethanol-induced dementia.

Edible insects are not only an excellent source of protein, but they also contain unsaturated fatty acids, dietary fiber, essential minerals, and vitamins [16]. In this study, in the results of the analysis of the composition of fatty acids in FPB, palmitic acid, oleic acid, and linoleic acid were the major fatty acids detected (Table 1). Consistent with our study, previous studies have reported that the essential amino acid and unsaturated fatty acid contents of *P. brevitarsis* larvae were higher than those of other edible insects such as *Tenebrio molitor* and *Allomyrina dichotoma* [18,19]. Human studies have reported a significantly reduced risk of cognitive decline and dementia in a population sample with high consumption of dietary unsaturated fatty acids such as PUFA and MUFA [21]. According to Nowakowski et al., edible insects such as silkworms and *Tenebrio molitor* can improve neuroinflammation, contributing to the prevention of Alzheimer’s disease (AD) and Parkinson’s disease [22]. *P. brevitarsis* has also been reported to inhibit neuroinflammation in lipopolysaccharide-induced BV-2 microglial cells [23]. Additionally, in the current study, the neuroprotective effect of FPB on H_2_O_2_ and ethanol-administered hippocampal HT22 cells was confirmed prior to the conducting of the animal study (Figure 1). Therefore, we hypothesized that FPB would be effective in the treatment of ethanol-induced cognitive dysfunction.

Well-established ethanol-induced learning and memory impairment models have been used for the evaluation of potential bioactive agents in the management of dementia. Previous studies have reported that consumption of ethanol can cause neurobehavioral symptoms such as anxiety and learning and memory impairment by direct toxicity on neurons [2]. These effects resemble the clinical symptoms of early AD and can be a cause of impairment in daily life. In this study, memory function was evaluated using the Y-maze, passive avoidance, and MWM tests. According to the results, behavioral disorders were observed in the ethanol group compared with the control group. However, results of the Y-maze and passive avoidance tests showed that treatment with FPB led to improvement of working memory and short-term memory. In addition, mice that consumed FPB spent a longer amount of time in the target zone (W zone) on the probe test and exhibited more rapid learning compared with the ethanol group (Figure 2). These results are consistent with those of a previous study, which reported that seizure behavior was alleviated in mice with trimethyltin (TMT)-induced cognitive deficits who were treated with *P. brevitarsis* larvae [13]. According to another study, treatment with silkworm, a representative edible insect, resulted in improved memory ability on the Y-maze and MWM tests in scopolamine-induced mice [24]. These findings suggested that treatment with FPB can improve learning and memory ability in mice with chronic ethanol-induced dementia.

Chronic consumption of alcohol can lead to the production of excessive ROS as the metabolism of EtOH in the liver can be a mechanism for the development of neurodegeneration [25]. Cellular oxidative stress states are caused by an imbalance between the production of ROS and antioxidant systems. Chronic consumption of alcohol can reduce the levels of endogenous antioxidants such as SOD and glutathione peroxidase (GPX), as well as the levels of GSH, catalase, and vitamins, increasing the severity of oxidative stress [26]. An increase in lipid peroxidation and protein oxidation reactions can be caused by high levels of oxidative stress, leading to the destruction of the cell membrane structure of the brain and increasing permeability [3]. As a result, increased permeability of the BBB can enable large molecules and harmful substances to reach the brain, causing damage to the nervous system [27]. Therefore, chronic consumption of alcohol places the brain in a state of excessive oxidative stress, which may contribute to the development of cognitive impairment. Previous studies have reported on the in vitro antioxidant activity and ROS inhibition effects of *P. brevitarsis* larvae in AML12 cells [12,28]. In addition, treatment with *P. brevitarsis* larvae resulted in reduced levels of ROS in mice with radiation-induced testicular injury [29]. According to a study reported by Lee et al., treatment with *P. brevitarsis* water extract resulted in improved cognitive function via the regulation of nuclear factor erythroid 2-related factor 2 (Nrf2)-mediated oxidative stress in the hippocampus of TMT-treated mice [13]. In addition, FPB contains an abundance of PUFAs, which have been reported to inhibit the generation of free radicals in traumatic brain injury rat models, thereby reducing oxidative stress [30]. Consistent with previous studies, in this study, the levels of MDA and SOD were regulated, and the levels of GSH were reduced in brain tissues after treatment with FPB, leading to restoration of ethanol-induced oxidative stress (Figure 3). These findings suggest that FPB can protect the brain from excessive oxidative stress induced by consumption of ethanol, thereby leading to improved cognitive function.

An association of cognitive decline resulting from chronic consumption of alcohol with the cholinergic system has been reported [8]. Loss of the neurotransmitter ACh due to degeneration of cholinergic neurons is a common symptom of dementia. Synthesis of ACh from choline and acetyl CoA occurs via ChAT and its decomposition into choline and acetate by AChE. According to Casamenti et al., a behavioral disorder related to decreased levels of ACh and increased activity of AChE was observed after consumption of ethanol for a period of three months in the cortex and hippocampus of rats [9]. In addition, a previous study reported that the decline in cholinergic function caused by long-term consumption of ethanol cannot be recovered even when ethanol consumption is stopped [31]. However, dietary PUFAs, which are an essential component of neuronal plasma membranes, can affect the formation and exocytosis of synaptic vesicles containing neurotransmitters [32]. Therefore, supplementation with dietary PUFAs may be beneficial for successful neurotransmission. In addition, the results for the polyunsaturated fatty acid (PUFA) content of FPB, including oleic acid, linoleic acid, and linolenic acid, were 357.07, 81.13, and 6.00 mg/100 g, respectively (Table 1). These results were significantly higher than those for raw *P. brevitarsis* larvae (9.44, 0.69, and 0.03 g/100 g, respectively) reported in a previous study [33]. Therefore, the protective effect of FPB on the cholinergic system can be attributed to the abundance of PUFAs in FPB as well as the reduction of ethanol-induced oxidative stress.

Chronic consumption of ethanol can lead to an imbalance in intracellular redox states, which can cause mitochondrial damage [4]. Mitochondrial damage and subsequent cell death have been reported as occurring early in the development of neurological disease [3]. An association of decreased outer and inner mitochondrial membrane potentials, which regulate the transport of energy substrates through β-oxidation, with the main cause of alcohol-induced mitochondrial dysfunction has been reported [34]. β-oxidation in damaged mitochondria caused by direct or indirect toxicity of alcohol can be a cause of imbalance in oxidative phosphorylation, resulting in leakage of oxidation products from the substrate and depletion of ATP [35]. Consequently, chronic consumption of ethanol can lead to mitochondrial dysfunction characterized by accumulation of ROS, production of ATP, and reduction of MMP within mitochondria. However, a previous study reported that treatment with protein extracts from *P. brevitarsis* resulted in the improvement of mitochondrial function via H_2_O_2_-induced reduction of MMP in C2C12 myoblast cells [11]. PUFAs are known to be involved in homeostasis of mitochondrial calcium, generation of ROS, and mitochondrial apoptosis, which can be beneficial in the prevention of diseases such as AD, myocardial infarction, diabetes, and obesity [36]. A previous study reported that consumption of dietary polyunsaturated fatty acid including omega-3 resulted in significantly improved mitochondrial respiration and ATP levels in brain tissue in aged rats [37]. In addition, omega-3 fatty acid supplementation had a protective effect on mitochondrial energy metabolism and oxidative stress in rats regularly administered L-tyrosine [38]. Likewise, we found that consumption of FPB led to the improvement of alcohol-induced mitochondrial dysfunction in the brain (Figure 5). Therefore, it can be assumed that the improvement of mitochondrial function observed in this study by treatment with FPB can be attributed to the abundance of PUFAs.

Ethanol-induced oxidative damage and mitochondrial dysfunction can cause changes to apoptosis-related proteins in mitochondria, leading to neuronal cell death [25]. Cells exposed to alcohol can become stressed due to various factors, including accumulation of ROS, damaged DNA, and various metabolic chemicals [1]. This can lead to the initiation of the intrinsic apoptosis pathway, which in turn leads to the activation of pro-apoptotic proteins, such as BCL-2 homologous antagonist killer (BAK) and BAX, and the suppression of anti-apoptotic proteins such as BCL-2 and BCL-extra-large (BCL-XL) [39]. The caspase pathway is then activated with the release of cytochrome c from mitochondria, leading to the induction of apoptosis. Excessive activation of caspase-3 is a characteristic of neurodegenerative diseases such as AD [39]. In addition, regulation of the AKT/GSK-3β pathway functions as a neuroprotectant through promotion of cell survival and inhibition of apoptosis [40]. Indeed, consumption of alcohol can induce dementia through apoptosis related to neuronal loss [41]. Treatment with *P. brevitarsis* protein extract has been reported to decrease the BAX/BCL-2 ratio, thereby exerting a protective effect against cytotoxicity in H_2_O_2_-induced C2C12 cells [11]. In addition, the results of pretreatment with *P. brevitarsis* larvae showed a reduction in the number of apoptotic nuclei in radiation-induced rat testicular germ cells [29]. In agreement with findings reported in previous studies, in this study, FPB exhibited anti-apoptotic activity through a decrease in the BAX/BCL-2 ratio and activation of the AKT/GSK-3β pathway in ethanol-administered mice (Figure 6). Therefore, it is presumed that brain neuronal death caused by ethanol was suppressed by the anti-apoptotic activity of FPB, thereby contributing to improvement of cognitive function.

An association of increased levels of pro-inflammatory cytokines with several neurological disorders related to learning and memory abilities has been reported [7]. Chronic consumption of alcohol can be a cause of neuroinflammation via activation of TLR-4 in microglial cells, which can directly or indirectly contribute to the development of brain damage and cognitive impairment [42]. TLR has an important function in inflammatory responses through activation of NF-κB and regulation of genes encoding chemokines, growth factors, and pro-inflammatory cytokines such as IL-1β and TNF-α [43]. Pascual et al. reported that ethanol can be a cause of neuroinflammation and anxiety-like behavioral disorders through activation of the TLR-4/NF-κB signaling pathway [43]. However, the anti-inflammatory activity of *P. brevitarsis* larvae as well as a variety of other edible insects has been attributed to their active proteins and peptides [6,18]. In addition, previous studies reported that LPS-induced inflammatory responses in RAW 264.7 macrophage and BV-2 microglia cells were improved by treatment with *P. brevitarsis* [23,44]. Treatment with *P. brevitarsis*-derived protein hydrolysate was also reported to result in the improvement of systemic inflammation through a decrease in the levels of cytokines, including IL-6, IL-17a, IL-1β, and TNF-α, in HFD-induced mice [45]. Treatment with a water extract of *P. brevitarsis* resulted in improved hippocampal neurodegeneration in rats with TMT-induced neuroinflammation [17]. Likewise, in this study, treatment with FPB resulted in down-regulated expression of TLR-4, p-IκB α, p-NF-κB, IL-1β, and TNF-α (Figure 7). These results provide evidence that FPB can be beneficial in ameliorating cognitive dysfunction through improvement of neuroinflammation by suppressing activation of the TLR-4/NF-κB pathway in ethanol-administered mice.

Neuroinflammation and oxidative stress can cause impairment of synapse function, affecting memory ability. In particular, high levels of TNF-α can cause impairment of synaptic plasticity through the disruption of synaptic signaling pathways associated with intra-cellular Ca^2+^ stores [46]. In addition, excessive oxidative stress can lead to loss of synaptic connections and information processing abilities, thereby causing a decline in learning and memory abilities [3]. Synaptic damage is characterized by abnormal sprouting and loss of plasticity, resulting in a failure to maintain synaptic connections, eventually leading to the development of neurodegeneration [47]. In addition, acute and chronic exposure to alcohol can affect synaptic plasticity in the striatum, neocortex, and hippocampus, which are involved in cognitive function [47]. SYP, a presynaptic vesicle protein, is an indicator of synaptic plasticity and density in the brain and has an essential function in the biogenesis of synaptic vesicles, release of neurotransmitters and endocytosis, and recycling of synaptic vesicles [48]. An association of GAP-43 with presynaptic neuronal outgrowth and axonogenesis and its increased expression during axon sprouting has been reported [49]. PSD-95, located beneath the postsynaptic membrane, has an essential function in synaptic development and synaptic activity [48]. Therefore, preventing the loss of synapse-related proteins may be a mechanism for improving cognitive function. Treatment with representative edible insects, *Gryllus bimaculatus* and *Oxya chinensis sinuosa*, was reported to result in the increased expression of PSD-95 in the brain tissue of rats with pentylenetetrazol-induced epilepsy [14]. According to a previous study, treatment with *Allomyrina dichotoma* larvae extract inhibited the decrease of postsynaptic proteins, including phospho-NMDA receptor (GluN)2A, GluN2B, and PSD-95, for the maintenance of the structure and function of hippocampal neurons in a hippocampal culture model [50]. These findings suggest that extracts derived from edible insects can influence the improvement of neuroplasticity. Similarly, decreased levels of synapse-related proteins were detected in the ethanol group; however, treatment with FPB resulted in significantly up-regulated expression of SYP and GAP-43 (Figure 8). Therefore, it is assumed that the protective effect of FPB against the loss of neuroplasticity proteins was helpful to the improved memory observed in the current study.

Chronic consumption of ethanol can be a cause of oxidative stress and inflammatory response, and it can affect the function of the BBB [7]. Disruption of BBB integrity due to increased BBB permeability can cause synaptic and neuronal dysfunction and is considered an early biomarker of cognitive decline [51]. The BBB is compacted by tight junctions (TJ) and junctional attachment molecule (JAM) proteins, such as claudins, occludins, and ZO-1, which regulate the intercellular diffusion of molecules [7,51]. Therefore, increased permeability resulting from damage to the BBB is associated with decreased expression of the TJ protein, which can ultimately lead to cognitive dysfunction. A previous study reported that treatment with representative edible insects, *Gryllus bimaculatus* and *Oxya chinensis sinuosa*, resulted in improvement of brain damage by regulating expression of claudin-5, occludin, ZO-1, and MMP-2 [14]. In addition, Ge et al. reported that treatment with *Tenebrio Molitor* resulted in increased intestinal barrier function through reinforcement of tight junction proteins such as ZO-1, ZO-2, and Mucin 3 in largemouth bass [52]. In this study, the function of the BBB was protected by FPB through increased expression of ZO-1, occludin, and claudin-1 in brain tissue of ethanol-administered mice (Figure 9). To date, research on the effect of FPB on improving function of the BBB has been limited; thus, comparison under the same conditions is difficult. However, several studies have reported on the antioxidant and anti-inflammatory activities of FPB [12,13,44]. Therefore, considering the previously reported results, the findings of this study demonstrated the beneficial effect of FPB on ethanol-induced neuroinflammation and oxidative stress, and it can be presumed that these effects contributed to the integrity of the BBB. Therefore, the protective effect of FPB against damage to the BBB is related to the restoration of cognitive function.

## 4. Materials and Methods

### 4.1. Chemials and Antibodies

Dulbecco’s Modified Eagle’s Medium (DMEM), calf serum (CS), penicillin, streptomycin, dimethyl sulfoxide (DMSO), MTT, and DCF-DA were purchased from Sigma-Aldrich Chemical Co. (St. Louis, MO, USA). A SOD kit was obtained from Dojindo Molecular Technologies (Rockville, MD, USA). Ethanol was purchased from Ethanol Supplies World Co., Ltd. (Jeonju, Republic of Korea). Dimethyl sulfoxide (DMSO), 2-thiobarbituric acid (TBA), trichloroacetic acid (TCA), o-phthaldialdehyde (OPT), acetylthiocholine, AChE, pyruvic acid, malic acid, mannitol, egtazic acid (EGTA), hydroxyethyl piperazine ethane sulfonic acid (HEPES), 5,5′,6,6′-tetrachloro-1,1′,3,3′-tetraethylbenzimidazolocarbocyanine iodide (JC-1), N,O-bis(trimethylsilyl)trifluoroacetamide(BSTFA), and all other reagents used were purchased from Sigma-Aldrich Chemical Co. (St. Louis, MO, USA).

The primary and secondary antibodies are listed below: ACh, AChE, SYP, PSD-95, GAP-43, TLR-4, NF-κB, IκB-α, IL-1β, TNF-α, BCL-2, BAX, caspase-3, ZO-1, occludin, claudin-1, and β-actin were purchased from Santa Cruz Biotech (Dallas, TX, USA). ChAT and Horseradish peroxidase (HRP)-conjugated anti-rabbit IgG secondary antibody were purchased from Cell Signaling Tech (Danvers, MA, USA). HRP-conjugated anti-mouse IgG secondary antibody purchased from Bio-Rad (Hercules, CA, USA).

### 4.2. Sample Preparation

The fermented *P. brevitarsis* larvae powder product used in the experiment was supplied by HMO Health Dream (Taean, Republic of Korea). To make the industrial value of *P. brevitarsis* larvae, they were fermented using hydrolyzate as follows. 100 g of *P. brevitarsis* larvae was dissolved in 1 L distilled water and hydrolyzed by proteases including papain and bromelain (Bision Corp., Seoul, Republic of Korea). Then, it was incubated with 2% (*v*/*v*). *B. subtilis* KCTC 1428BP (1 × 10^5^ CFU/mL) for 48 h at 30 °C. The FPB was evaporated under reduced pressure and lyophilized. Prepared samples were stored at −20 °C until further use.

### 4.3. Cell Culture

HT22 cells that are derived from mouse hippocampal neuronal cells were supplied in December 2021 by the Department of Anatomy of the College of Veterinary Medicine, Gyeongsang National University, Jinju, Republic of Korea. Cells were cultured on DMEM containing 10% (*v*/*v*) CS, 100 units/mL penicillin-streptomycin in a 5% CO_2_ incubator.

### 4.4. Determination of Cell Viability in HT22 Cells

The cell proliferation effect of FPB on HT22 cells was determined using an MTT assay. HT22 cells were seeded in 96 well-plates (1 × 10^4^ cells/well) and incubated for 24 h. The cells were pretreated with various concentrations of FPB and 200 μM vitamin C. After 30 min, 500 mM ethanol was treated and incubated for 24 h. Additionally, 200 μM H_2_O_2_ was treated for 3 h. Then, cells were incubated with MTT reagent (5 mg/mL) for 3 h at 37 °C, and the resulting formazan product was dissolved in DMSO. The cell viability was measured by using a microplate reader (Epoch 2, BioTek Instruments, Inc., Winooski, VT, USA) at 570 nm (detection wavelength) and 630 nm (reference wavelength).

### 4.5. Determination of Intracellular ROS Production in HT22 Cells

The intracellular oxidative stress reduction effect of FPB was evaluated using a DCF-DA assay. HT22 cells were seeded in 96 well black plates (1 × 10^4^ cells/well) and incubated for 24 h. The cells were pretreated with various concentrations of FPB and 200 μM vitamin C. After 30 min, 500 mM ethanol was treated and incubated for 24 h. Additionally, 200 μM H_2_O_2_ was treated for 3 h. Then, cells were incubated with DCF-DA reagent (10 μM) for 50 min at 37 °C. The cell ROS production was detected by using a fluorescence microplate reader (Infinite 200, Tecan Co., San Jose, CA, USA) at 485 nm (excitation wavelength) and 530 nm (emission wavelength).

### 4.6. Animals and Treatments

Male C57BL/6 mice (4 weeks old, *n* = 15; *n* = 5 for mitochondrial tests, *n* = 7 for in vivo and ex-vivo tests, *n* = 3 for Western blot assay) were purchased from Samtako (Osan, Republic of Korea). The mice were placed in groups consisting of 3–4 animals per cage and housed under the conditions of 12/12 h light/dark cycles at 22 ± 2 °C. Mice were assigned to 4 groups: control group, ethanol group, and FPB 50 (50 mg/kg of body weight) and FPB 100 (100 mg/kg of body weight) groups. FPB and ethanol groups were treated with FPB or drinking water by oral gavage, respectively. After 30 min, 5 g/kg body weight of 25% (*v*/*v*) ethanol was orally administered except for the control group. After 8 weeks, mice were anesthetized by exposure to CO_2_ gas, and then blood samples and brain tissues were collected. All animal study procedures were approved by the Institutional Animal Care and Use Committee (IACUC) of Gyeongsang National University guidelines (Certificate No. GNU-220710-M0079) on 10 July 2022.

### 4.7. Behavioral Tests

#### 4.7.1. Y-Maze Test

The Y-maze test was conducted as described previously [53]. The Y-maze (length, 33 cm; height, 15 cm; width, 10 cm) consists of three arms that form a Y shape with equal angles. Mice were placed at the end of one arm and allowed to explore the maze. The movement of mice in Y-maze was recorded by a smart 3.0 video tracking system (Panlab, Barcelona, Spain) for 8 min.

#### 4.7.2. Passive Avoidance Test

The passive avoidance test was performed as reported previously [53]. This experiment was performed using a chamber (width, 410 mm; depth, 210 mm; height, 300 mm) consisting of a bright and dark region, and these regions are separated by a central door. In training tests, mice were allowed to acclimatize in a dark chamber for 1 min and then move unimpeded between dark and light chambers for 2 min. However, the moment they entered the dark zone, the mice received an electric shock (0.5 mA, 3 s). After 24 h, in the test trial, the step-through latency time to the dark zone was measured at a maximum of 300 s.

#### 4.7.3. Morris Water Maze (MWM) Test

The MWM test was performed as explained previously [53]. MWM pool consists of a stainless steel circular pool randomly divided into four sections (E, W, S, and N) of equal area. The pool was filled with water dissolved in white non-toxic ink, and the water temperature was maintained at 20 ± 2 °C. The test was conducted for 6 days, including a visible training test (day 1), hidden test (days 2–5), and probe test (day 6). A platform was placed in the center of the W zone, and its position was not changed during the test. In the hidden test, the latency time for mice to reach the platform was recorded at a maximum of 60 s. The trials were conducted 4 times a day for 4 consecutive days. In the probe test (day 6), the time spent in the W zone was recorded for 60 s after removing the platform.

### 4.8. Preparations of Brain Tissues for Biochemical Analysis

After behavioral evaluation, brain tissues were removed and rinsed with normal saline. Then, phosphate-buffered saline (PBS) equivalent to 10 times the tissue weight was added and homogenized with beads. Each brain tissue homogenate was separated into three portions for different biochemical analyses. Aliquots of resulting brain homogenates were stored at −80 °C until utilization.

### 4.9. Measurement of Antioxidant Systems

#### 4.9.1. MDA Content

Brain tissues were homogenated with phosphate-buffered saline (PBS) and centrifuged at 2500× *g* for 10 min. The obtained supernatant was mixed with 1% phosphoric acid and 0.67% TBA and reacted at 95 °C for 1 h. To determine the MDA content, the absorbance of the reactant was measured at 532 nm.

#### 4.9.2. Reduced GSH

Brain tissues were homogenated with 10 mM phosphate buffer containing 1mM EDTA (pH 6.0) and centrifuged at 10,000× *g* for 15 min. The obtained supernatant was mixed with 5% metaphosphoric acid and centrifuged at 2000× *g* for 2 min. The obtained supernatant was reacted with 0.26 M Tris-HCl (pH 7.6), 0.65 N NaOH, and OPT (1 mg/mL). After 15 min, to determine the reduced GSH level, the fluorescence of the reactant was measured at 320 nm (excitation wavelength) and 420 nm (emission wavelength).

#### 4.9.3. SOD Level

Brain tissues were homogenized with PBS and centrifuged at 400× *g* for 10 min. The pellet was mixed with 1 × cell extraction buffer containing 10% SOD buffer, 0.4% (*v*/*v*) triton X-100, and 200 μM phenylmethane sulfonylfluoride (PMSF). Then, the mixture was centrifuged at 10,000× *g* for 10 min. To confirm the SOD level, supernatants were used with a commercial SOD kit (Dojindo Molecular Technologies, Rockville, MD, USA).

### 4.10. Measurement of ACh Content and AChE Activity

ACh content and AChE activity in brain tissue were detected as described previously [54]. Brain tissues were homogenized with PBS and centrifuged at 14,000× *g* for 30 min. The supernatant was reacted with alkaline hydroxylamine reagent (3.5 M NaOH and 2 M hydroxylamine-HCl) and had 0.5 M HCl and 0.37 M FeCl_3_-6H_2_O added to it. Then, the absorbance was immediately measured at 540 nm.

To evaluate the AChE activity in brain tissues, the supernatant was mixed with 50 mM sodium phosphate buffer (pH 8.0) and incubated at 37 °C for 15 min. Then, Ellman’s reaction mixture (1 mM 5,5′-dithio-bis(2-nitrobenzoic acid) and 0.5 mM acetylthiocholine iodide in 50 mM sodium phosphate buffer (pH 8.0) was added to it and it was incubated at 37 °C for 10 min. The absorbance was read at 405 nm.

### 4.11. Measurement of Mitochondrial Function

#### 4.11.1. ROS Level

Isolation of mitochondria from brain tissues was performed as described previously [53]. The isolated mitochondria extract from brain tissues was mixed with a KCl-based respiration buffer containing 125 mM KCl, 2 mM KH_2_PO_4_, 20 mM HEPES, 1 mM MgCl_2_, 500 μM EGTA, 2.5 mM malate, and 5 mM pyruvate. Then, the mixture was reacted with 25 μM of DCF-DA for 20 min at room temperature. After, the fluorescence intensity was measured at 485 nm (excitation wavelength) and 530 nm (emission wavelength).

#### 4.11.2. MMP

The isolated mitochondria extract from brain tissues was incubated with 1 mM JC-1 in mitochondrial isolation buffer (MI; 215 mM mannitol, 75 mM sucrose, 0.1% BSA and 20 mM HEPES sodium salt (pH 7.0)) containing 5 mM pyruvate and 5 mM malate. The mixture was incubated at room temperature for 20 min in dark conditions. Then the fluorescence intensity was detected at 530 nm (excitation wavelength) and 590 nm (emission wavelengths).

#### 4.11.3. ATP Content

ATP content was determined using a commercial ATP bioluminescence assay kit (Promega, Madison, WI, USA) according to the manufacturer’s protocol. The ATP content was calculated according to a standard curve.

### 4.12. Western Blot Analysis

Brain tissues were homogenized in ice-cold RIPA buffer (Cell signaling Tech, Danvers, MA, USA) containing 1% protease inhibitor cocktails (Thermo Fisher Scientific, Rockford, IL, USA). Brain tissues lysates were centrifuged at 13,000× *g* for 10 min, and the protein concentration of supernatant was identified using a BSA assay (Bradford reagent, Bio-Rad, CA, USA). The samples quantified in equal amounts (25–50 μg) of total protein were separated on SDS polyacrylamide gel and transferred onto a PVDF membrane (Millipore, Burlington, MD, USA). Membranes were blocked with 5% skimmed milk and incubated overnight with respective primary antibodies. Then, the membranes were reacted with the corresponding secondary antibodies for 1 h. The intensity of protein bands was visualized by using an enhanced chemiluminescence detection reagent (ECL, Translab, Daejeon, Republic of Korea) and measured using an iBright CL1000 imager (Thermo Fisher Scientific, Rockford, IL, USA). β-actin was used as a loading control.

### 4.13. Fatty Acid Composition of FPB

After placement of 2 g of FPB in a cylindrical filter paper (Toyo Roshi Kaisha, Ltd., Tokyo, Japan), ether was added, and extraction was performed using the Soxhlet extraction method for 16 h to obtain crude fat, followed by addition of 0.5 N KOH-methanol to the extracted crude fat for 15 h. Methyl esterification was then performed at 90 °C for 40 min. BF_3_-methanol was then added, followed by heating at 90 °C for 2 min, and cooling was then allowed. Hexane and saturated sodium chloride solution were then added, followed by centrifuging to obtain a hexane layer. BSTFA was added to the hexane layer and analysis was performed using gas chromatography (GC, Shimadzu GCMS-TQ8030, Shimadzu, Tokyo, Japan). A SUPELCOWAX 10 column (60 m × 0.32 mm, Supelco, Bellefonte, PA, USA) was used, and analysis of the injector, column oven, and detector temperatures was performed at 250 °C, 260 °C, and 280 °C, respectively. Nitrogen was used as the carrier gas, and the split ratio was set to 30:1.

### 4.14. Statistical Analysis

All data were showed as mean ± SD. Statistical analysis was analyzed using one-way analysis of variance (ANOVA) followed by Duncan’s multiple range test by SAS program (Ver. 9.4 SAS Institute, Cary, NC, USA). Statistical difference (*p* < 0.05) of each group was shown by different lowercase letters with superscripts.

## 5. Conclusions

In summary, this study demonstrated that FPB can protect against ethanol-induced cognitive dysfunction. These findings suggest that FPB can not only enhance the antioxidant system, mitochondrial function, and cholinergic system but can also be effective against cognitive behavioral disorders caused by the consumption of alcohol through suppression of the AKT and NF-κB pathways. In addition, up-regulated neuroplasticity and BBB function were attributed to the ameliorating effects of FPB on neuroapoptosis and neuroinflammation. Therefore, the findings of this study demonstrated the potential for the use of FPB as a preventive strategy to ameliorate the effects of ARD.

## Figures and Tables

**Table 1:** Fatty acid composition of protein hydrolysate from *Protaetia brevitarsis* larvae fermented by *Bacillus subtilis* (FPB).

Fatty Acids	Amount (mg/100 g)	% of Total Fatty Acids (%)
Myristic acid (C14:0)	5.30 ± 0.00	0.79 ± 0.00
Palmitic acid (C16:0)	143.80 ± 0.70	21.43 ± 0.13
Palmitoleic acid (C16:1)	48.67 ± 0.23	7.25 ± 0.04
Stearic acid (C18:0)	24.17 ± 0.06	3.60 ± 0.01
* Oleic acid (C18:1, n-9, cis)	357.07 ± 2.35	53.22 ± 0.27
* Linoleic acid (C18:2, n-6, cis)	81.13 ± 0.50	12.10 ± 0.09
* Linolenic acid (C18:3, n-3)	6.00 ± 0.00	0.90 ± 0.00
Arachidic acid (C20:0)	4.80 ± 0.00	0.72 ± 0.00
Total fatty acid	670.9 ± 1.30	100

The results were presented as the mean ± SD (*n* = 3). * Essential amino acids.

## Data Availability

The data presented in this study are available on request from the corresponding author.
